# Behavioral Changes and Determinants of Preventive Oral-Health Practices Among Chinese Dental Patients: A Hospital-Based Cross-Sectional Study in Guangzhou

**DOI:** 10.3390/dj14070460

**Published:** 2026-07-21

**Authors:** Jiali Yu, Zhe Ji, Rui Jiang, Rafiqul Islam, Xiang Li, Ermin Nie

**Affiliations:** 1 First Affiliated Hospital, Sun Yat-sen University, 58 Zhong Shan Road 2nd, Guangzhou 510080, China; yujli7@mail.sysu.edu.cn (J.Y.); jizh7@mail.sysu.edu.cn (Z.J.); jiangr9@mail.sysu.edu.cn (R.J.); hpyylx@163.com (X.L.); 2 Department of Restorative Dentistry, Faculty of Dental Medicine, Hokkaido University, Kita 13, Nishi 7, Kita-ku, Sapporo 060-8586, Japan; rony12cdc@den.hokudai.ac.jp

**Keywords:** preventive oral health, behavior change, dental caries, oral disease, dental treatment

## Abstract

**Background**: Preventive oral-health behaviors are crucial for reducing the incidence of dental caries, but there is still little data on whether Chinese dental patients continue to practice these behaviors. This study investigated self-reported changes in oral-health practices after treatment and explored factors associated with the adoption of preventive behaviors. **Methods**: A cross-sectional questionnaire-based survey was carried out in a hospital in Guangzhou with 246 dental patients. The frequency of brushing both before and after treatment, interdental cleaning, dietary modifications, attendance at follow-up, receiving and understanding preventive advice, and following recommendations were reported. Changes in brushing behavior were analyzed, and logistic regression models were used to evaluate factors associated with preventive behaviors. **Results**: The proportion of participants brushing ≥2/day increased after treatment (7.7%, *p* < 0.05). Daily interdental cleaning remained low (23.2%), while 61.0% reported dietary modification and 53.3% attended follow-up. In multivariable models adjusting for all covariates, most sociodemographic factors were not significantly associated with preventive behaviors. Male participants had lower odds of brushing ≥2/day. Understanding of preventive recommendations was the most consistent predictor, with lower understanding associated with reduced flossing, follow-up, and adherence. Receipt and format of advice were not independently associated with outcomes after adjustment. Periodontal treatment was associated with lower follow-up compared with restorative care. **Conclusions**: Preventive oral-health behaviors improved modestly after treatment, but overall adherence remained limited. Clear understanding of preventive advice was the strongest determinant of behavior, while sociodemographic factors and advice format showed no independent effects. Lower follow-up attendance among periodontal patients highlights the need for improved patient communication and recall strategies.

## 1. Introduction

Oral health is a vital component of overall health and quality of life, influencing essential functions such as nutrition, communication, and social interaction [1]. Globally, oral diseases lead to serious health and economic burdens, resulting in a significant reduction in quality of life [2]. Oral diseases—including dental caries, periodontal disease, and traumatic dental injuries—exert a negative impact on oral health and overall quality of life [3]. Although oral diseases can be prevented and controlled, there is a great difference in the treatment effect of diseases because people have different levels of knowledge about oral diseases, and their compliance with oral-health behaviors varies [2]. An essential element to achieving good oral health is the adoption of efficient and effective oral-health behaviors [2]. The adoption and maintenance of oral hygiene habits have been described by a number of behavioral models, such as the Health Belief Model, the Transtheoretical Model, and self-efficacy-based theories [4,5].

Preventive oral-health behaviors such as regular tooth-brushing, interdental cleaning, dietary modifications, and routine dental check-ups are fundamental in controlling and reducing the incidence and progression of oral diseases [1,6]. Previous research indicates that interventions focused on patient beliefs, motivation, and self-regulation can improve plaque control and oral hygiene quality [7,8]. At the same time, clinical outcomes vary and appear to depend largely on patients’ engagement and their level of understanding [8]. In addition, previous studies have also shown that effective dentist–patient communication improves comprehension, confidence, adherence, and patient satisfaction [9]. For this reason, communication is widely regarded as a core element of quality oral-health care.

Importantly, oral-health care is best conceptualized as an integrated continuum where dental treatment and preventive care are inseparable and mutually reinforcing components. Preventive care, including oral hygiene instruction and reinforcement, should be embedded within treatment episodes to maximize long-term oral-health outcomes [10]. However, many dental care systems worldwide continue to operate in a predominantly treatment-oriented model, focusing primarily on addressing existing disease rather than systematically integrating prevention throughout care delivery [11]. Such a model risks missing critical opportunities to support meaningful and sustained improvements in oral hygiene behaviors, which are essential for reducing disease burden at the population level.

In China, especially in large cities like Guangzhou, although awareness of oral health has increased in recent years [12], there is still a large gap in the maintenance of preventive behavior after treatment. Our previous studies have explored the knowledge, attitudes and practices related to caries prevention among patients [12], as well as the current status and challenges of dentists in the implementation of caries risk assessment [13]. These studies provide preliminary data for understanding the dentist–patient factors affecting dental disease prevention and control but have yet to fully elucidate whether patients modify their daily behaviors following dental interventions or how they adhere to the preventive recommendations from dental practitioners.

Therefore, this study aimed to evaluate changes in oral-health behaviors before and after dental treatment among Chinese dental patients and to identify sociodemographic, behavioral, communication-related, and treatment-related factors associated with the adoption of preventive oral-health behaviors.

## 2. Materials and Methods

### 2.1. Study Design

This cross-sectional questionnaire study evaluated self-reported changes in oral-health behaviors after treatment. This study received ethical approval (approval no:2025/573, dated 15 August 2025) from the IEC for clinical research and animal trials of the First Affiliated Hospital of Sun Yat-sen University. Data collection was conducted from September to November 2025, for a total of three months. The study was designed and reported in accordance with the Strengthening the Reporting of Observational Studies in Epidemiology (STROBE) guidelines for observational studies. The study utilized Chinese language questionnaires that were distributed to dental patients in Guangzhou. The questionnaires were originally developed in English and subsequently translated into Chinese. All participants voluntarily provided written informed consent. Participation was voluntary and all data were collected anonymously to ensure confidentiality.

### 2.2. Sample Size Calculation

The sample size for this cross-sectional study was calculated using the following single-proportion formula:

$$n=\frac{Z^{2}p\left(1-p\right)}{d^{2}}$$

Assuming a behavioral change prevalence of 80% based on findings from our preliminary pilot study, a confidence interval of 95% (*Z* = 1.96) and a margin of error of 5% (*d* = 0.05) were applied, resulting in a required sample size of 246. To account for the possibility of missing data, the sample size was increased by 2% to a final number of 250 participants.

### 2.3. Participant Selection

A convenience sampling method was used to recruit eligible patients attending the dental hospital during the study period. Eligible criteria were patients attending the dental hospital, aged 18 years or older, who had previously completed dental treatment, and who were able to understand and complete the questionnaire. The exclusion criteria were patients with severe systemic diseases, with cognitive impairment, and who were unable to respond to the questionnaire. A total of 250 patients responded to the survey. Of these, 4 questionnaires were excluded due to incomplete data, leaving 246 fully completed responses for analysis.

### 2.4. Questionnaire Structure and Content

The questionnaire included demographic information such as age, sex (male/female), highest educational attainment (primary, high school, college, postgraduate or above), and monthly household income (<3000; 3000–5000; 5000–8000; >8000 RMB). Participants indicated the time elapsed since they completed their most recent dental treatment, categorized into within 3 months, 3–6 months, 6–12 months, or >12 months. Participants reported their brushing frequency before and after treatment using four categories: occasionally, once daily, twice daily, more than twice daily. For analytic purposes, brushing was additionally dichotomized into ≥2 times/day (twice or more) vs. ≤1 time/day (once or occasionally). Interdental cleaning was assessed using three categories (daily/occasionally/rarely) and dichotomized as daily vs. not daily for regression models. Participants also reported whether they modified their diet to prevent future dental problems (yes/no), or attended a post-treatment professional check-up (yes/no). Participants indicated whether they received preventive advice on caries control (yes/no) or referral to a scheduled follow-up visit after completion of treatment rather than ongoing visits required as part of active treatment. Among those receiving advice, the format was recorded as verbal, written material, digital/video content, or practical demonstration. Participants who reported receiving preventive advice were additionally asked to indicate how clearly they understood the recommendations and the extent to which they followed them. Understanding was originally collected as a categorical variable with two response options: very clear and somewhat clear. Adherence was recorded by asking participants to indicate whether they followed the recommendation fully or followed the recommendation partially. These simplified categorical measures were used to minimize respondent burden and improve feasibility in a clinical survey setting. The questionnaire was reviewed by experts and pilot-tested among dental patients to ensure clarity and relevance. However, these brief measures may not fully capture the complexity of patient understanding and adherence behaviors. Participants rated their belief that daily habits can prevent dental problems on a 5-point scale (1 = not at all, 5 = completely). In addition, participants were asked to report the types of dental treatment they had received during their most recent course of care. Treatment types included restorative procedures (dental filling, inlay, crown restoration, and root canal treatment) and periodontal therapy. For analysis, treatments were categorized as restorative treatment only, periodontal treatment only, or combined restorative and periodontal treatment.

### 2.5. Validation

Before data collection, the questionnaire was reviewed and tested through several validation steps. The draft questionnaire was evaluated by dental research experts to evaluate content validity and sufficiently addressed the study question. After that, a small group of dental patients participated in a pilot test of the first version to evaluate the reliability and clarity of the questions. Participants provided feedback on the questionnaire’s length, language, and response options during the pilot stage. In order to increase comprehension and response accuracy, small changes were made to the question phrasing and the response scale based on this feedback.

### 2.6. Statistical Analysis

Descriptive statistics were used to summarize sociodemographic characteristics, oral-health behaviors, and preventive advice variables and to compare uptake of preventive behaviors across treatment categories (restorative only, periodontal only, and combined treatment). Continuous variables are presented as mean ± standard deviation, and categorical variables as frequencies and percentages. Changes in brushing frequency before and after treatment were evaluated in two ways: (i) Four-category brushing variable: the McNemar–Bowker test was used to assess distributional shifts across the four brushing categories. (ii) Dichotomized brushing (≥2/day vs. ≤1/day): the McNemar test was used to evaluate paired binary changes, with net improvement calculated along with a 95% confidence interval. To identify factors associated with oral-health behaviors after treatment, logistic regression models were constructed for the following outcomes: brushing ≥2/day after treatment, daily interdental cleaning, modified diet, return for professional check-up, (among advice recipients) fully following dentist recommendations. For full-sample models, covariates included sex, age, education, income, time since treatment, receipt of preventive advice (yes/no), and belief score. For models restricted to participants who received preventive advice, the variable “received advice” was replaced by advice format and level of understanding. Age and belief score were treated as continuous variables. Adjusted odds ratios (aORs), 95% confidence intervals (CIs), and *p*-values were reported. All covariates were specified a priori based on theoretical and clinical relevance, and no data-driven variable selection procedures (e.g., stepwise selection) were applied. Multicollinearity among independent variables was assessed using variance inflation factors (VIFs), and no evidence of significant multicollinearity was identified. Missing data were handled using complete-case analysis, whereby observations with missing values were excluded from the regression models. Multivariable logistic regression models were additionally used to examine associations between treatment type and preventive behaviors, adjusting for age and sex, with restorative treatment serving as the reference category. All analyses were conducted using Python version 3.12 (statsmodels package) and cross-validated using standard statistical procedures.

## 3. Results

### 3.1. Participant Characteristics

A total of 246 participants were analyzed (mean age 30.66 ± 10.37 years). Among the participants, 60.2% were women. Education levels were as follows: primary, 4.9%; high school, 6.9%; college, 77.2%; and postgraduation or above, 11.0%. Monthly salary was as follows: <3000 RMB, 5.7%; 3000–5000 RMB, 14.2%; 5000–8000 RMB, 26.0%; and >8000 RMB, 54.1%. Time since completion of treatment was as follows: within 3 months, 28.9%; 3–6 months, 6.1%; 6–12 months, 8.1%; and >12 months, 56.9%. The sociodemographic characteristics of the study population are summarized in Table 1.

### 3.2. Oral-Health Behavior

Changes in tooth-brushing frequency before and after treatment are presented in Table 2A. Before treatment, brushing frequency was as follows: once, 21.1%; twice, 68.3%; ≥3/day, 8.1%; and occasionally, 2.4%. After treatment, it was as follows: once, 14.2%; twice, 74.4%; ≥3/day, 9.8%; and occasionally, 1.6%. Interdental cleaning behavior, dietary modification, and follow-up attendance after treatment are shown in Table 2B. Interdental cleaning frequency was as follows: daily, 23.2%; occasionally, 60.6%; and rarely, 16.3%. A proportion of 61.0% reported modifying their diet to prevent future cavities, and 53.3% attended a post-treatment professional check-up (follow-up visit after completion of treatment).

### 3.3. Preventive Advice and Adherence

Overall, 79.7% of the participants reported receiving preventive advice. Among advice recipients, the format was as follows: verbal, 57.1%; written, 19.9%; digital/video, 14.8%; and practical demonstration, 8.2%. Understanding of the advice was very clear for 48.5% or somewhat clear for 51.5%. The extent of following recommendations was fully for 43.4% and partially for 55.6%. The most frequently reported barriers were forgetting for 32.7%, lack of time for 21.4%, high cost for 18.4%, perceiving advice as unnecessary for 15.8%, and instructions being too complicated for 11.7%. Details regarding preventive advice, clarity of understanding, adherence, and reported barriers are summarized in Table 3.

### 3.4. Paired Change in Brushing

When all four brushing categories (occasionally, once daily, twice daily, ≥3/day) were examined, the Bowker test indicated a significant asymmetry in the before–after distribution (χ^2^ = 12.76, df = 5, *p* < 0.05), demonstrating a significant overall shift toward higher brushing frequency after treatment (Table 4A). When brushing was dichotomized as ≥2 times/day versus ≤ 1 time/day, 26 participants improved while 7 worsened. The McNemar test showed a significant (*p* < 0.05) net improvement (+7.7) in brushing behavior (Table 4B).

### 3.5. Adjusted Associations with Preventive Behaviors

The adjusted logistic regression models examining factors associated with preventive oral-health behaviors after treatment are presented in Table 5A. In models including all prespecified covariates, most sociodemographic variables, including age, education level, income, and time since treatment, were not significantly associated with preventive behaviors after adjustment. Male participants had significantly lower odds (*p* < 0.05) of brushing ≥2 times per day compared with female participants. However, no consistent associations between sex and other preventive behaviors were observed. Receipt of preventive advice was not independently associated with most behavioral outcomes after adjustment. Similarly, belief scores regarding the role of daily habits in preventing dental problems were not significantly associated with preventive behaviors in the multivariable models. Understanding of preventive recommendations emerged as the most consistent factor associated with adherence-related outcomes. Lower levels of understanding were associated (*p* < 0.05) with reduced odds of daily interdental cleaning, returning for professional follow-up, and fully following dentist recommendations. However, understanding was not significantly associated with brushing frequency or dietary modification.

The adjusted associations among participants who reported receiving preventive advice are shown in Table 5B. In these models, which additionally included advice format and level of understanding, understanding remained significantly associated (*p* < 0.05) with all adherences-related outcomes. Participants who reported lower clarity of understanding had significantly reduced odds of daily interdental cleaning, follow-up attendance, and adherence to recommendations. In contrast, advice format was not significantly associated with preventive behaviors after adjustment, although some non-significant trends were observed. Sociodemographic variables, including education and income, were not independently associated with adherence outcomes in this subgroup.

### 3.6. Preventive Behavior Uptake by Treatment Type

When preventive behaviors were examined descriptively by type of dental treatment, differences in uptake were observed across treatment groups (Table 6). Improvements in tooth-brushing frequency to ≥2 times per day were modest overall but slightly higher among patients receiving periodontal treatment (14.0%) or combined treatment (14.3%) compared with those receiving restorative treatment only (9.7%). Daily flossing remained limited across all treatment groups, ranging from 18.6% among periodontal patients to 28.6% among those receiving combined care. Dietary modification was reported by more than half of the participants in all treatment groups, with the highest proportion observed among patients receiving periodontal treatment (67.4%). Professional follow-up attendance varied substantially by treatment type, being highest among patients receiving combined restorative and periodontal care (85.7%) and lowest among those receiving periodontal treatment alone (37.2%).

### 3.7. Association Between Type of Dental Treatment and Preventive Behaviors

As shown in Table 7, treatment type was not significantly associated with improvement in tooth-brushing frequency to ≥2 times per day, daily flossing, or dietary modification. Compared with patients who received restorative treatment only, those who underwent periodontal treatment showed no independent association with brushing improvement (adjusted odds ratio [aOR] = 1.05; 95% CI 0.11–10.41) or daily flossing (aOR = 0.53; 95% CI 0.08–3.33). Similarly, no significant association (*p* > 0.05) was observed between combined treatment and these preventive behaviors. In contrast, a significant association (*p* < 0.05) was observed for professional follow-up attendance. Patients who received periodontal treatment were less likely to report post-treatment professional follow-up compared with those who received restorative treatment (aOR = 0.10; 95% CI 0.01–0.89). The distribution of treatment types was as follows: restorative treatment (n = 196), periodontal treatment (n = 43), and combined treatment (n = 7).

## 4. Discussion

This cross-sectional study assessed changes in oral-health behaviors following dental treatment and identified the factors associated with the adoption of preventive behaviors among Chinese patients. In the present study, a significant improvement in brushing frequency was observed after treatment, which indicates that dental visits can serve as teachable moments where patients become more motivated to adopt oral-health practices [14]. This suggests that dental treatment and provided instructions may serve as effective motivators for behavior change [15]. However, despite a highly educated sample and relatively high income levels, daily interdental cleaning remained low. Previous research has shown that Chinese patients rarely use dental floss due to reduced manual dexterity, cost, and limited understanding of its benefits [16,17]. Previous studies have suggested that dental care utilization may often be problem-oriented, with visits influenced by symptoms such as pain or discomfort [18,19]. In addition, more than half of the participants reported dietary modifications aimed at preventing future caries and had returned for professional dental check-ups after treatment. These behaviors reflect some awareness of preventive oral-health measures among participants [20]. However, the results indicate that many patients did not adopt a wide variety of preventative behaviors or participate in routine follow-up care. Understanding oral-health information is crucial for patients to follow preventive recommendations. Clear communication can help patients make informed decisions about their own oral health, and it has been linked to increased confidence, adherence, and satisfaction with treatment. For this reason, previous studies highlight the need for patient-centered approaches in dentist–patient communication [21].

Although most patients in this study stated that they received preventive advice and believed the information was clear, full adherence was comparatively low. The most commonly employed format was verbal instructions, with written, digital, and demonstration-based approaches being less common. This limited use of multiple formats may help explain why many participants followed recommendations only partially and why forgetting and lack of time were commonly reported barriers. Previous studies have shown that delivering information through multiple formats such as written or visual reinforcement is associated with better retention and adherence than verbal communication alone [21,22]. Using additional communication tools, including written instructions, digital reminders, or chairside demonstrations, may help patients apply preventive advice more consistently in daily oral-health care.

The paired analysis showed a clear improvement in tooth-brushing frequency following dental treatment. An increase was observed in the proportion of patients brushing at least twice daily, suggesting that more participants reported adopting recommended oral hygiene practices after treatment. The absolute increase of 7.7 percentage points indicates a modest but measurable change in brushing behavior. Similar associations between regular tooth-brushing and the prevention of periodontal disease and dental caries have been reported in previous studies [23,24]. In addition, the shift toward higher brushing frequency was evident across categories, supporting the consistency of this behavioral change. Patients who reported more frequent dental visits and brushing also appeared more engaged in daily oral care and more attentive to maintaining their oral health [25]. These observations underscore the relevance of the dentist–patient relationship in encouraging positive oral-health behaviors [25].

In the present study, most sociodemographic variables, including age, education, and income, were not significantly associated with preventive behaviors after adjustment. This suggests that behavioral adherence may be influenced less by socioeconomic characteristics and more by individual perceptions and behavioral factors. Previous research has similarly shown that oral-health behaviors are not solely determined by demographic variables but are strongly influenced by psychological and behavioral determinants such as self-efficacy and attitudes [26]. A key finding of this study is that understanding of preventive recommendations was the most consistent factor associated with adherence-related behaviors. Participants with poorer understanding were less likely to engage in interdental cleaning, attend follow-up visits, and adhere to recommendations. This aligns with evidence that effective dentist–patient communication and clarity of information are critical for improving adherence and treatment outcomes. In addition, studies have demonstrated that patients often fail to recall or fully understand information provided during consultations, which can negatively affect adherence to oral-health instructions [22,27,28]. In contrast, among participants who received preventive advice, the format of delivery was not independently associated with adherence outcomes. These findings suggest that improving the clarity and quality of communication may be more important than the specific mode of delivery. Therefore, strategies that enhance patient understanding such as simplified explanations, reinforcement, and patient-centered communication approaches may be more effective in promoting sustained behavioral change [27].

A significant finding of this study is the variation in preventive behavior uptake based on the type of dental treatment received. Patients who underwent combined restorative and periodontal care demonstrated the highest rates of professional follow-up attendance. This suggests that more complex or comprehensive treatment plans may enhance engagement with oral health, potentially increasing the patient’s perceived necessity of maintenance [29]. Another finding of this study is that patients who received periodontal treatment alone were significantly less likely to attend post-treatment professional follow-up compared with those receiving restorative care. This is of particular clinical concern, as periodontal disease is a chronic condition that requires ongoing maintenance and regular professional care to prevent disease recurrence and progression [30]. Several potential explanations may account for this finding. Periodontal treatment outcomes are often less immediately noticeable to patients once symptoms such as pain or swelling have resolved, which may reduce the perceived need for continued care. In addition, periodontal maintenance typically requires repeated visits over time, and previous research has shown that patient adherence to supportive periodontal therapy is often suboptimal and influenced by multiple factors including awareness, perceived need, and psychological or behavioral barriers [31,32]. Limited patient understanding of the chronic nature of periodontal disease may further contribute to lower follow-up rates. Importantly, evidence indicates that adherence to supportive periodontal care is strongly associated with improved long-term clinical outcomes, including reduced disease progression and tooth loss [33]. Therefore, the low follow-up attendance observed in this study highlights a critical gap in post-treatment care. Targeted strategies such as improved patient education, clear communication regarding disease progression, and structured recall or reminder systems may be needed to enhance adherence among periodontal patients.

This study has a number of limitations that should be noted. First, a causal relationship between preventive advice and behavioral change cannot be inferred due to the cross-sectional methodology. Second, oral-health behaviors were assessed using self-reported measures, which may introduce recall bias and social desirability bias and could result in overestimation of positive behaviors. In particular, pre-treatment behaviors were collected retrospectively based on participant recall, whereas post-treatment behaviors reflected current self-reported practices. This difference in measurement timing limits direct comparison and precludes causal inference regarding behavioral changes following dental treatment. Third, the results may not be as applicable to the larger Chinese population due to the convenience sampling technique and the sample’s high level of education and age. Moreover, the study was carried out in a single facility, which might not accurately represent regional differences in dental care practices. Furthermore, for some outcomes such as daily interdental cleaning, the relatively limited number of events may result in a lower events-per-variable ratio in the regression models. This may increase the risk of overfitting and affect the stability of the estimated associations. Therefore, these findings should be interpreted with caution. The relatively small number of participants in the combined treatment group, as well as the limited size of the periodontal group, may have reduced the statistical power and contributed to the wide confidence intervals observed in the analyses stratified by treatment type. Therefore, these findings should be interpreted with caution. In addition, the study did not include other important determinants of health behavior, such as oral-health literacy, self-efficacy, and motivation, which have been shown to influence preventive practices. Future studies incorporating validated multi-dimensional instruments are needed to better understand these factors. Notwithstanding these limitations, the study possesses distinct strengths as it investigated various sociodemographic and communication-related factors that influence preventive behavior, patient perceptions of preventative advice, and behavioral changes both before and after treatment.

## 5. Conclusions

Among Chinese dental patients in Guangzhou, self-reported oral-health behaviors appeared to show modest improvement following dental treatment, particularly brushing frequency. Other preventive measures, such as frequent interdental cleaning and full adherence to professional advice, were still not widely followed. Clear understanding of preventive recommendations emerged as the most consistent determinant of adherence, whereas sociodemographic factors and the format of advice delivery were not independently associated with behavioral outcomes. Patients receiving periodontal treatment were less likely to attend follow-up care, highlighting a critical gap in long-term disease management. These findings highlight the importance of strengthening patient-centered communication and reinforcing preventive education to support sustained oral-health behavior change.

## Figures and Tables

**Table 1:** Sociodemographic characteristics of the study participants.

Variable	Category	*n*	%
Age, years	Mean ± SD	30.66 ± 10.37	—
Sex	Female	148	60.2
	Male	98	39.8
Education	Primary	12	4.9
	High school	17	6.9
	College	190	77.2
	Postgraduation	27	11.0
Monthly salary (RMB)	<3000	14	5.7
	3000–5000	35	14.2
	5000–8000	64	26.0
	>8000	133	54.1
Time since treatment	Within 3 months	71	28.9
	3–6 months	15	6.1
	6–12 months	20	8.1
	>12 months	140	56.9

**Table 2:** (**A**) Self-reported tooth-brushing frequency before and after dental treatment among participants. (**B**) Self-reported preventive oral-health behaviors after treatment, including interdental cleaning frequency, dietary modification to prevent future caries, and attendance at professional post-treatment check-ups.

(**A**)			
Time	Category	*n*	%
Before	Once	52	21.1
	Twice	168	68.3
	≥3/day	20	8.1
	Occasionally	6	2.4
After	Once	35	14.2
	Twice	183	74.4
	≥3/day	24	9.8
	Occasionally	4	1.6
(**B**)			
Variable	Category	*n*	%
Interdental cleaning	Daily	57	23.2
	Occasionally	149	60.6
	Rarely	40	16.3
Modified diet to prevent caries	Yes	150	61.0
Post-treatment professional check-up	Yes	131	53.3

**Table 3:** Characteristics of preventive advice received by participants.

Variable	Category	*n*	%
Advice format	Verbal	112	57.1
	Written	39	19.9
	Digital/video	29	14.8
	Practical demonstration	16	8.2
Understanding	Very clear	95	48.5
	Somewhat clear	101	51.5
Followed recommendations	Fully	85	43.4
	Partially	109	55.6
Main barriers	Simply forgot	64	32.7
	Lack of time	42	21.4
	High cost	36	18.4
	Advice felt unnecessary	31	15.8
	Too complicated	23	11.7

**Table 4:** Paired comparison of tooth-brushing frequency before and after dental treatment. (**A**) Four-category paired comparison of brushing frequency before and after treatment (occasionally, once daily, twice daily, ≥3 times/day). Distributional differences were assessed using the Bowker test of symmetry. (**B**) Dichotomized paired comparison of brushing frequency (≤1 time/day vs. ≥2 times/day). Paired changes were assessed using the McNemar test.

(**A**)				
Before /After	Occasionally	Once	Twice	≥3/day
Occasionally	1	0	2	3
Once	0	31	20	1
Twice	2	4	156	6
≥3/day	1	0	5	14
(**B**)				
	After ≤1/day	After ≥2/day		Total
Before ≤1/day	32	26		58
Before ≥2/day	7	181		188

Bowker test: χ^2^ = 12.76, df = 5, *p* < 0.05. McNemar: *p* = 0.001; net improvement = +7.7.

**Table 5:** (**A**) Adjusted logistic regression models examining associations between participant characteristics and preventive oral-health behaviors after treatment. (**B**) Adjusted logistic regression models among participants who received preventive advice, assessing associations between advice format, clarity, and adherence-related outcomes.

(**A**)										
Variable	Brushing ≥2/Day aOR (95% CI)	*p *-Value	Flossing Daily aOR (95% CI)	*p *-Value	Dietary Change aOR (95% CI)	*p *-Value	Follow-Up aOR (95% CI)	*p *-Value	Following Recommendations aOR (95% CI)	*p* -Value
Age	1.03 (0.99–1.07)	0.121	1.01 (0.98–1.05)	0.403	1.01 (0.99–1.04)	0.330	1.00 (0.97–1.02)	0.783	0.98 (0.95–1.02)	0.312
Male (vs. Female)	0.30 (0.14–0.67)	0.003	1.71 (0.91–3.24)	0.097	0.89 (0.51–1.54)	0.671	0.96 (0.54–1.71)	0.898	0.97 (0.52–1.80)	0.926
Education	1.08 (0.56–2.10)	0.814	1.20 (0.67–2.16)	0.536	0.82 (0.51–1.32)	0.414	1.16 (0.72–1.87)	0.541	0.86 (0.51–1.44)	0.559
Income	0.98 (0.63–1.51)	0.919	0.98 (0.69–1.40)	0.924	0.81 (0.59–1.11)	0.187	0.96 (0.70–1.31)	0.775	0.79 (0.57–1.11)	0.178
Time since treatment	0.90 (0.67–1.21)	0.501	0.98 (0.77–1.24)	0.844	0.84 (0.69–1.03)	0.099	0.89 (0.72–1.10)	0.280	1.01 (0.80–1.27)	0.950
Received advice	1.20 (0.19–7.65)	0.845	0.72 (0.15–3.54)	0.684	1.53 (0.43–5.49)	0.513	0.96 (0.26–3.52)	0.947	4.91 (0.49–49.68)	0.178
Understanding	1.02 (0.41–2.55)	0.959	0.39 (0.20–0.78)	0.008	0.91 (0.50–1.67)	0.766	0.36 (0.19–0.66)	0.001	0.28 (0.15–0.52)	<0.001
Belief score	0.78 (0.54–1.12)	0.182	1.07 (0.81–1.42)	0.630	1.02 (0.79–1.31)	0.873	0.94 (0.73–1.21)	0.637	0.94 (0.72–1.23)	0.655
(**B**)										
Variable	Flossing Daily aOR (95% CI)	*p *-Value		Follow-Up aOR (95% CI)		*p *-Value	Following Recommendations aOR (95% CI)			*p *-Value
Age	1.01 (0.98–1.05)	0.340		1.00 (0.97–1.03)		0.866	0.99 (0.96–1.02)			0.353
Male	1.64 (0.89–3.05)	0.115		0.92 (0.52–1.60)		0.759	1.04 (0.57–1.91)			0.889
Education	1.17 (0.71–1.95)	0.530		1.09 (0.66–1.82)		0.730	1.15 (0.69–1.93)			0.580
Income	1.03 (0.67–1.60)	0.880		1.01 (0.66–1.56)		0.970	1.05 (0.67–1.64)			0.820
Time since treatment	1.02 (0.67–1.55)	0.920		1.05 (0.70–1.59)		0.810	0.98 (0.64–1.51)			0.920
Advice format	1.46 (0.82–2.60)	0.190		1.44 (0.82–2.54)		0.200	1.60 (0.87–2.92)			0.130
Understanding	0.38 (0.20–0.76)	0.006		0.36 (0.20–0.66)		0.001	0.30 (0.16–0.55)			<0.001
Belief score	1.18 (1.01–1.38)	0.039		1.21 (1.03–1.42)		0.019	1.36 (1.15–1.62)			<0.001

Abbreviations: aOR: adjusted odds ratio; CI: confidence interval; *p* < 0.05 was considered statistically significant.

**Table 6:** Preventive behavior uptake by type of dental treatment.

Outcome	Restorative	Periodontal	Combined
Brushing improvement	9.7%	14.0%	14.3%
Daily flossing	24.0%	18.6%	28.6%
Diet change	59.7%	67.4%	57.1%
Follow-up attendance	55.6%	37.2%	85.7%

**Table 7:** Association between type of dental treatment and adoption of preventive oral-health behaviors (restorative: n = 196; periodontal: n = 43; combined: n = 7).

Outcome	Treatment Comparison	Adjusted OR (aOR)	95% CI	*p* Value
Improved brushing (≥2/day)	Periodontal vs. restorative	1.05	0.11–10.41	0.969
	Combined	0.70	0.08–6.20	0.749
Daily flossing	Periodontal vs. restorative	0.53	0.08–3.33	0.499
	Combined	0.75	0.14–4.08	0.737
Dietary modification	Periodontal vs. restorative	1.48	0.29–7.54	0.640
	Combined	1.05	0.23–4.82	0.955
Professional follow-up	Periodontal vs. restorative	0.10	0.01–0.89	0.040
	Combined	0.21	0.03–1.77	0.150

## Data Availability

The data presented in this study are available on request from the corresponding author. The data are not publicly available due to the presence of potentially identifiable information and restrictions imposed by the institutional ethics committee.
